# Association between Long-Term Exposure to Particulate Matter Air Pollution and Mortality in a South Korean National Cohort: Comparison across Different Exposure Assessment Approaches

**DOI:** 10.3390/ijerph14101103

**Published:** 2017-09-23

**Authors:** Ok-Jin Kim, Sun-Young Kim, Ho Kim

**Affiliations:** 1 Department of Public Health Science, Graduate School of Public Health, Seoul National University, Seoul 08826, Korea; nadia331@snu.ac.kr; 2 Department of Cancer Control and Population Health, Graduate School of Cancer Science and Policy, National Cancer Center, Goyang 10408, Korea

**Keywords:** cohort, exposure assessment, long-term exposure, mortality, particulate matter

## Abstract

Increasing numbers of cohort studies have reported that long-term exposure to ambient particulate matter is associated with mortality. However, there has been little evidence from Asian countries. We aimed to explore the association between long-term exposure to particulate matter with a diameter ≤10 µm (PM_10_) and mortality in South Korea, using a nationwide population-based cohort and an improved exposure assessment (EA) incorporating time-varying concentrations and residential addresses (EA1). We also compared the association across different EA approaches. We used information from 275,337 people who underwent health screening from 2002 to 2006 and who had follow-up data for 12 years in the National Health Insurance Service-National Sample Cohort. Individual exposures were computed as 5-year averages using predicted residential district-specific annual-average PM_10_ concentrations for 2002–2006. We estimated hazard ratios (HRs) of non-accidental and five cause-specific mortalities per 10 µg/m^3^ increase in PM_10_ using the Cox proportional hazards model. Then, we compared the association of EA1 with three other approaches based on time-varying concentrations and/or addresses: predictions in each year and addresses at baseline (EA2); predictions at baseline and addresses in each year (EA3); and predictions and addresses at baseline (EA4). We found a marginal association between long-term PM_10_ and non-accidental mortality. The HRs of five cause-specific mortalities were mostly higher than that of non-accidental mortality, but statistically insignificant. In the comparison between EA approaches, the HRs of EA1 were similar to those of EA2 but higher than EA3 and EA4. Our findings confirmed the association between long-term exposure to PM_10_ and mortality based on a population-representative cohort in South Korea, and suggested the importance of assessing individual exposure incorporating air pollution changes over time.

## 1. Introduction

Many cohort studies, mostly performed in North America and Europe, have found that long-term exposures to particulate matter (PM) are associated with mortality [1,2,3]. In Asia, a few recent studies have examined this association, but these findings were inconsistent [4,5,6,7,8,9,10]. There has been an increasing research interest in the air pollution effects in Asian countries where PM concentrations are relatively high and the exposed population is exceptionally large [11,12,13,14].

Exposure assessment that represents spatially and temporally varying air pollution is particularly important in Asia. Many cohort studies have assessed individual-level air pollution concentrations computed over one or a few years at baseline, or prior to baseline, because of limited monitoring data and/or address information. Large cohort studies of PM in the U.S. and Europe used average concentrations that were limited to relatively shorter time periods because either cohort inception had occurred before the initiation of spatially-extensive regulatory monitoring networks or the project-based monitoring campaigns had only been carried out for limited periods [1,15]. The approach adopted for those studies assumed consistent spatial distributions of air pollution over time. However, this assumption may not hold in Asian countries that have experienced rapid economic development focusing on some urban areas over recent decades. In addition, individual-level concentrations were estimated at residential addresses at one fixed-time point, mostly at baseline in many previous studies, resulting from unavailable residential mobility information [1,16,17]. That approach may also poorly represent long-term exposure in Asian countries, and the impact of exposure measurement error on health analysis could be large.

A recently created population-representative national cohort in South Korea provides a unique opportunity to investigate the association between long-term air pollution and mortality, incorporating residential history information. South Korea achieved a universal coverage of health insurance for the entire population in 1989. To utilize the enormous health and medical service database for public health research, the National Health Insurance Service (NHIS) created the NHIS-National Sample Cohort (NHIS-NSC), in 2014 [18]. This cohort sampled about one million people from the National Health Insurance database in 2002 and included their follow-up data for 12 years through 2013. The cohort provides district-level individual addresses updated annually and rich information on demographic and socio-economic characteristics for the subset of the cohort who underwent health screening.

A recently-developed national-scale air pollution prediction model, and an approach for computing population-representative exposure, also allow the investigation of long-term association of exposure to air pollution with mortality in South Korea. This point-wise prediction model was the first attempt to estimate air pollution concentrations at any location in South Korea [19]. A follow-up study suggested an approach to compute district-level population-representative air pollution concentrations, which could be applied to epidemiological studies, using administrative health data with district-level addresses [20]. These methods facilitate assessing national-scale associations of air pollution in South Korea, including approximately 40% of districts in the year of 2010 without any regulatory monitoring sites.

This study aimed to investigate the association between long-term exposure to PM_10_ and non-accidental and cause-specific mortality, using a population-based nationwide cohort in South Korea. In addition, we compared the associations from three exposure assessment approaches depending on the incorporations of temporally-variant PM_10_ concentrations and/or residential mobility history.

## 2. Methods

### 2.1. Study Population

We used information on approximately 57.5% of the NHIS-NSC individuals (585,696 people) who participated in health screening, fully covered by NHIS. The screening service is offered to employees at all ages and all other citizens aged 40 years or older biannually, with extended screening for those at the ages of 40 and 65 considered as biologically significant ages over a lifetime. These individuals provided extended biological and socio-demographic information including anthropometry, health behavior such as smoking and underlying diseases through questionnaires and biological examinations. To retain sufficiently prolonged exposure periods that might impact on mortality, we restricted our study population to 314,305 people who had survived and participated in health screening for the first five years of the study period from 2002 to 2006. Figure S1 shows our exclusion criteria and the numbers of individuals excluded for each exclusion process. We excluded those who were younger than the age of 20 or older than 65 at baseline, were severely disabled, had no information for cause of death, or did not have follow-up data including addresses for two years or more. The final study population included 275,337 people (87.6% of 314,305).

This study was approved by the institutional review board of the Seoul National University (IRB no. E1605/003-004). 

### 2.2. Individual- and Area-level Covariates

For individual covariates, we included smoking status, frequency of alcohol use, frequency of physical activity, body mass index (BMI), and co-morbidity of cardiovascular disease, hypertension, and diabetes at the first health screening, within the first five years for 2002–2006. In addition, we used three area-level covariates corresponding to each individual’s residential district in 2002. The three covariates comprised the percentage of the elderly (≥65 years), educational attainment equal to or higher than high school from the 2000 Census [21], and the gross regional domestic product (GRDP) taken from general national statistics for 2005 [22]. We categorized some individual- and area-level covariates to represent non-linear relationships with mortality.

### 2.3. Mortality

The NHIS-NSC provides information on cohort individual mortality linked to death certificate data taken from Statistics Korea. We chose non-accidental and five cause-specific deaths occurring since 2007 using the International Classification of Diseases, 10th Reversion (ICD-10); non-accidental (A00–R99), cardiovascular disease (I00–I99), cerebrovascular disease (I60–I69), respiratory disease (J00–J99), cancer (C00–C97), and lung cancer (C33–C34) mortality. These deaths have been associated with particulate matter air pollution in previous studies [23,24]. Because the date of death was recorded using the year and month of death, we assigned the 15th day of the month as the day of all deaths to calculate survival time.

### 2.4. Exposure Assessment for Long-Term PM_10_ Concentration

For individual-level long-term PM_10_ concentrations, we used residential district-specific annual average PM_10_ concentrations for 2002–2006 estimated from a previously developed prediction model and an area-averaging approach. The spatial prediction model was developed in a universal kriging framework including summary predictors of more than 300 geographical variables and spatial correlation based on air quality regulatory monitoring data in South Korea [19]. The regulatory monitoring network included 185–261 monitoring sites for 2000–2006 in South Korea. Hourly PM_10_ concentrations were quantified by the beta-ray absorption method [25]. Geographical variables that largely contributed to the prediction model included characteristics of land use, demography, and emissions [19]. Subsequent work provided an approach to estimate district-level population-representative PM_10_ concentrations which allowed us to assess air pollution effects using administrative health data with area-level individual addresses, such as that derived from the NHIS-NSC, in South Korea [20]. Based on these two previous studies, we predicted annual average concentrations of PM_10_ at 83,463 centroids of residential census output areas in South Korea each year from 2002 to 2006, and computed an average in each district.

We computed 5-year average PM_10_ concentrations from 2002 through 2006, as individual long-term exposures to PM_10_, for NHIS-NSC individuals using four exposure assessment (EA) approaches as summarized in Table S1. These four approaches differ in their use of the incorporation of yearly updated PM_10_ concentrations and residential addresses: prediction and address in each year (EA1), prediction in each year and address at baseline (EA2), prediction at baseline and address in each year (EA3), and prediction and address at baseline (EA4) (see equations in the supplemental materials). Whereas EA1 represents temporally-varying exposures and addresses, other approaches are based on temporally-constant exposures and/or addresses at baseline for one year in 2002. Baseline exposures and addresses were commonly applied to long-term exposures and addresses in previous cohort studies because of limited available data [16,26,27]. For EA2 and EA4, we assumed that PM_10_ at baseline were consistent over time for 5 years. For EA3 and EA4, we assumed that people did not move. The split into the exposure and the event periods helped us avoid exposure assignment being dependent on follow-up duration. A previous study in California (U.S.A) showed much higher mortality risks of PM_2.5_ components than those in previous studies, because decreased concentrations in recent years were assigned to individuals with long follow-ups [28].

### 2.5. Statistical Analysis

We used the Cox proportional hazards model to estimate hazard ratios (HRs) and 95% confidence intervals (CIs) of non-accidental and cause-specific mortality per 10 µg/m^3^ increase in PM_10_ concentration. The survival time of each person was calculated in days from 1 January 2002, to the date of death, to the end of the study period on 31 December 2013, or to the drop-out date resulting from moving abroad.

We investigated the association between long-term exposure to PM_10_ and mortality using four progressively-adjusted models. We specified different baseline hazards by sex and age. We included long-term PM_10_ concentration only to model 1. In model 2, we added individual characteristics such as income, type of health insurance, smoking status, alcohol consumption, exercise, and obesity. Model 3 additionally included underlying diseases for hypertension, diabetes, and cardiovascular disease. Finally, we added all three area-level variables in model 4 as our primary model. Pearson’s correlation coefficients between all covariates were less than 0.7, indicating little evidence of collinearity (result not shown). We used model 4 to compare the association using our primary EA approach, EA1, with those using three other EA approaches. Comparing EA1 with EA2 and EA3 allowed us to assess the differences derived from temporally-invariant PM_10_ and residential addresses, respectively.

We performed several sensitivity analyses to examine the robustness of our results. First, we extended the exposure period to the end of the study. We computed a 12-year average using predicted annual average concentrations for the entire study period from 2002 through 2013 at a baseline residential address, regardless of when death occurred, as shown in previous studies [1,4,9]. Because we computed 12-year averages even for those who died during the follow-up period, we did not consider moving addresses and used baseline addresses only. This restriction would help us avoid the impact of assigning recent PM_10_ concentrations to those who survived. Secondly, we used PM_10_ measurements instead of predictions and investigated the association. Using hourly PM_10_ concentrations measured at approximately 250 air quality regulatory monitoring sites for 2002–2006, we calculated a 5-year average in each district [29]. This analysis was restricted to 119–142 (48.6–57.3%) districts in which there was at least one monitoring site. Thirdly, we considered a time-dependent Cox proportional hazards model. In this model, HR of mortality was estimated by combining all HRs estimated from one-year time windows between 2002 and 2006. Finally, we also explored HRs for long-term concentrations of PM_10_ using random intercept model while incorporating random effects for subject’s residential district at baseline to reflect the spatial dependency of mortality across people within each district.

All statistical analyses were carried out in SAS (version 9.4; SAS Institute Inc., Cary, NC, USA) and R (version 3.1.2, R Core Team 2014, Vienna, Austria), using the “survival” and “coxme” package (R Core Team 2014, Vienna, Austria).

## 3. Results

### 3.1. Study Population and Long-Term PM_10_ Concentration

Table 1 summarizes individual characteristics and mortality of 275,337 NHIS-NSC individuals across quintiles of long-term PM_10_ concentrations, defined as 5-year averages calculated using residential district-specific annual average PM_10_ concentrations at individual addresses from 2002 to 2006 (EA1). The average follow-up time was 4268 days (Standard Deviation (SD): 444 days). From 2007 through 2013, 4720 people (1.7%) died. There were 3796 non-accidental deaths (80.4% of all deaths), with cancer as the most common cause of death (n = 2179; 0.8%). Individual characteristics were generally similar across quintiles of PM_10_ concentrations, with some variations in age and contextual confounders. People exposed to higher long-term PM_10_ concentrations tended to be younger and lived in the districts with smaller elderly populations, higher educational attainment, and greater GRDP.

The average long-term PM_10_ concentration in 275,337 NHIS-NSC individuals, based on EA1, was 56.0 (SD = 6.5), which was slightly higher than the air quality standard of 50 µg/m^3^ in South Korea. Across the four EAs, the average long-term PM_10_ concentration was similar but the SD was larger in EA3 and EA4, which were based on one-year concentrations at baseline (mean = 55.9–57.6, SD = 6.5–9.2) (Table S2). Measurement-based long-term PM_10_ showed a larger mean (60.4) than the four prediction-based EA approaches, with a relatively wide SD (8.6). Long-term concentrations between the four different EAs were highly correlated (Pearson’s correlation coefficient = 0.87–0.96).

### 3.2. Association between Long-Term PM_10_ and Mortality

We found a marginal association between long-term PM_10_ concentrations based on EA1 and non-accidental mortality (HR: 1.05, 95% CI: 0.99–1.11) in our primary model, model 4 (Table 2). The HRs of all cause-specific mortality were greater than one, except lung-cancer mortality, although none were statistically significant (Table 2). The HRs of cerebrovascular mortality (1.14, 0.93–1.39) and respiratory mortality (1.19, 0.91–1.57) were greater than those of cardiovascular mortality (1.02, 0.90–1.16) and cancer mortality (1.02, 0.95–1.10). There was an increasing trend of HRs with progressively added individual- and area-level variables, reaching the highest HRs in model 4 (Table 2).

### 3.3. Comparisons of the Associations across Four EAs

**Figure 1:**
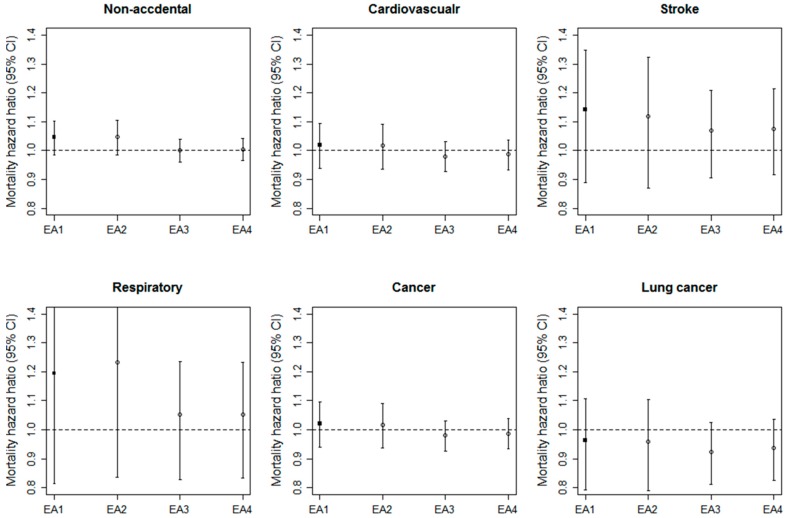
Hazard ratios (HRs) and 95% confidence intervals (CIs) of non-accidental and cause-specific mortality for an increase of 10 μg/m^3^ in the long-term PM_10_ concentration defined by four different exposure assessment (EA) approaches after adjusting for sex, age, income, smoking, alcohol use, obese, exercise, and co-morbidity of cardiovascular disease, cerebrovascular disease, and diabetes, district-level percent of high school education completed or more, percent of the elderly, and gross regional domestic product [EA1: prediction and address in each year, EA2: prediction in each year and address at baseline, EA3: prediction at baseline and address in each year, EA4: prediction and address at baseline].

Figure 1 shows HRs and 95% CIs of non-accidental and cause-specific mortality from model 4 across different individual long-term concentrations of PM_10_, estimated from four EAs. For non-accidental mortality, the HR for EA1 was similar to that for EA2 using time-varying PM_10_ concentrations with baseline addresses, and higher than those for EA3 and EA4 using constant PM_10_ concentrations at baseline. The pattern of HRs based on EA1, which was similar to EA2 and different from EA3 and EA4, was generally consistent across all five cause-specific mortality factors.

### 3.4. Sensitivity Analyses

Long-term PM_10_ concentrations, based on 12-year averages at baseline addresses, gave generally consistent results with higher HRs than those in our primary analysis using EA1 (Table S4). The HRs based on measurement-based PM_10_ were slightly lower than those of EA1 (Table S5). In the time-dependent Cox proportional hazards models, the HRs were lower for all mortality except for cancer mortality (Table S6). Finally, addition of a random intercept yielded similar HRs and narrow 95% CIs (Table S7).

## 4. Discussion

Using a population-representative national cohort, we found a marginal association between long-term exposure to PM_10_ and non-accidental mortality. Although we did not find an association for cause-specific mortality, the risk estimates of cerebrovascular and respiratory mortality were higher than those of cardiovascular and cancer mortality. This association was weak when we did not incorporate time-varying PM_10_ concentrations.

To our knowledge, this is the first study to report the association between long-term exposure to air pollution and mortality on a national scale in South Korea. The large cohort including one million people sampled from the entire population provided a rare opportunity to examine an association generalizable to the population. In addition, we used improved individual exposure estimates incorporating temporally-varying PM_10_ concentrations and residences over a 12-year period. There have been only a few cohort studies of air pollution in which updated address information is available [28,30,31]. Good characterization of long-term exposures is particularly important since mortality effects of air pollution have largely relied on durations of exposure periods and temporal trends of air pollution [28,32]. Table S8 summarizes large spatial-scale cohort studies of PM_10_ exposure and mortality based on several areas or an entire country. In general, long-term exposure to PM_10_ was marginally or significantly associated with total or non-accidental mortality, as shown in our study [1,7,26]. Likewise, the estimated risks for respiratory mortality were larger in Dutch and English cohorts than for cardiovascular mortality, consistent with our results, although other national studies in France and Germany showed the reverse pattern. While large cohort studies in North America and Europe observed greater risk estimates of cardiovascular mortality for long-term exposure to PM_2.5_ than those of respiratory or non-accidental mortality [2,33], this pattern may not be consistent for PM_10_. In South Korea, national-scale PM_2.5_ regulatory monitoring data were available from 2015 and future studies should investigate whether the association of PM_10_ found in our study differs from that of PM_2.5_.

Our findings indicate that the characterization of spatial and temporal changes in air pollution concentrations over time is more important for assessing long-term exposure than an improvement of spatial accuracy representing moving addresses. In the comparison of the associations across different exposure assessment approaches, EA2 using temporally-updated annual PM_10_ concentrations predicted at baseline addresses gave consistent risk estimates with those of EA1 using updated PM_10_ concentrations and updated addresses from each year. In contrast, EA3 and EA4 based on the application of one-year PM_10_ averages at baseline to all years showed lower risk estimates than those of EA1 and EA2, regardless of whether changing addresses were incorporated. Different from our results, a national cohort study in the U.S. found that time-varying PM_2.5_ for 2000–2008 gave similar risk estimates of mortality to those of time-constant baseline exposure in 2000 [27]. This indicates systematic changes in air pollution concentrations over the years with a constant spatial rank of concentrations within each year, as shown in the Harvard Six Cities Study, U.S. [34]. However, the spatial distribution of air pollution may not be consistent even over a decade in countries, such as South Korea, which experienced dramatic economic development affecting spatial redistribution of air pollution sources and their emissions over time. The observed spatial distribution of district annual averages in South Korea varied substantially across three different years within the 2002–2013 period (Figure S2). The minor impact of residential mobility on the association found in our study may be driven through relatively infrequent residential moving. A Canadian cohort study found consistent associations between the associations of long-term exposure to PM_10_ for the 1984–2006 period, based on tracking vs. baseline addresses, and the authors interpreted this pattern as resulting from less frequent residential moving [30]. Only approximately 10% of the population moved between different districts in South Korea between 2003 and 2013, with decreasing movement in more recent years [35]. The average duration of living in the same district, according to the 2005 Census, was about 7.7 years [36]. However, our address information limited to districts may have prevented us from identifying residential mobility within a district and assessing the impact of mobility at a fine spatial scale.

Although our study was based on large population and refined individual long-term exposure estimates, we did not find associations between PM_10_ and cause-specific mortality. There are several plausible explanations. First, our study population comprised relatively healthy people. Our study population included people aged 20–64 years at baseline. Most cohort studies have included individuals aged 40–89 years who were more at risk of dying [7,37,38]. In our review for national-scale cohort studies, non-accidental mortality rates were relatively low with 1.3% to 23.6% compared to the rate of 1.7% in our cohort (Table S8). The Dutch and German cohort studies with mortality rates of 9% and 18%, respectively, reported that long-term exposures to PM_10_ were significantly associated with increased mortality [26,38]. In contrast, a Chinese cohort study with low mortality rate of 1.3% also reported a statistically significant association [6]. This association, however, could result from large mean and standard deviation of PM_10_ in Shenyang, China compared to those in South Korean. In addition, district-level addresses would have resulted in reduced exposure variability across individuals and imprecise health effect estimates in subsequent health analyses. In our additional analysis, we found increased standard deviation of predicted annual average PM_10_ concentrations with increased means across 83,000 census output area centroids. This pattern indicates decreased exposure variability when averaged in each district. The exposure measurement error could also increase or decrease biases in health effect estimates [31,39].

Our study has several limitations that need to be addressed in future studies. Our study estimated individual air pollution exposures using individual addresses available at the district level in the NHIS-NSC database to assess national-scale associations. Future studies should investigate the impact of exposure measurement error resulting from the use of coarse address data on health effects analysis by substituting fine scale address data from project-based cohorts. Secondly, we did not include additional potential confounders for the relationship between air pollution and mortality, such as education and occupational exposure. Project-based cohort studies including these confounders should be used to verify and confirm our results. Thirdly, our study used predicted PM_10_ concentrations instead of measurements to include the entire country in the analysis. Uncertainty in exposure prediction might have affected our findings. Future studies should investigate the influence of prediction uncertainty on bias and imprecision in health effect estimates. In addition, although previous studies reported stronger associations of long-term exposure to PM_2.5_ with mortality than those of PM_10_, national-scale PM_2.5_ regulatory monitoring data in South Korea are available from 2015 which is behind our study period based on our cohort, NHIS-NSC. Future studies using recent cohorts should investigate the association of PM_2.5_. Lastly, long-term health effects of air pollution may vary across time periods over lifetime. Thus there may be a critical exposure period that gave particularly large health effects of air pollution, which is an important research topic for future research.

## 5. Conclusions

Based on a population-based cohort in South Korea, our study confirmed the association between PM_10_ and non-accidental mortality. Our findings also suggested the importance of incorporating changes in ambient air pollution concentrations over time, particularly in countries where socio-economic changes have occurred rapidly.

## Figures and Tables

**Table 1:** Summaries of demographic characteristics (%) of 275,337 National Health Insurance Service- National Sample Cohort (NHIS-NSC) subjects for 2002–2013 across quintiles of their long-term PM_10_ concentrations defined as 5-year averages calculated using annual average PM_10_ concentrations at residential addresses for 2002–2006 in South Korea.

Characteristics		Number of Subjects	PM_10_ Concentration (µg/m^3^)					
			Total (39.2–72.1)	Quintile 1 (39.2–49.3)	Quintile 2 (49.3–53.9)	Quintile 3 (53.9–59.5)	Quintile 4 (59.5–61.9)	Quintile 5 (61.9–72.1)
Total		275,337	100.0	19.8	20.2	19.6	20.5	19.9
Sex	Male	149,735	54.4	53.3	54.2	54.9	55.0	54.5
	Female	125,602	45.6	46.7	45.8	45.1	45.0	45.5
Age at baseline (years)	20–24	24,943	9.1	7.8	9.5	10.1	9.3	8.6
	25–29	31,843	11.6	8.5	10.4	12.9	13.3	12.6
	30–34	32,092	11.7	9.9	11.2	11.8	12.9	12.6
	35–39	35,837	13.0	12.4	13.2	12.7	13.6	13.2
	40–44	43,769	15.9	16.8	15.6	15.6	16.0	15.4
	45–49	35,816	13.0	13.7	12.8	13.2	12.6	12.6
	50–54	27,692	10.1	11.4	10.0	10.0	9.3	9.6
	55–59	22,072	8.0	9.4	8.1	7.4	7.1	8.1
	60–64	21,273	7.7	10.1	9.1	6.3	5.9	7.2
Income ^1^ (%)	<20	39,246	14.3	14.6	15.5	13.3	14.0	13.9
	20–50	72,764	26.4	26.6	27.2	24.9	26.4	27.0
	50–80	93,126	33.8	33.2	34.1	33.0	34.5	34.4
	>80	70,201	25.5	25.6	23.2	28.8	25.2	24.8
Type of health insurance	Self-insured	94,475	34.3	37.0	35.1	32.3	32.1	35.2
	Employee-insured	180,041	65.4	62.6	64.6	67.5	67.6	64.6
	Medical aid	821	0.3	0.4	0.4	0.3	0.3	0.2
Smoking	Non-smoker	179,428	65.2	67.5	66.3	64.9	63.3	64.0
	Ex-smoker	13,092	4.8	4.1	4.4	5.3	5.1	4.8
	Current smoker	82,817	30.1	28.4	29.3	29.8	31.6	31.2
Alcohol use (>3 times/week)		25,935	9.4	10.5	9.3	8.7	9.1	9.5
Exercise (<3 times/week)		227,960	82.8	82.3	83.8	82.2	83.0	82.7
Obese (BMI > 25.0)		83,894	30.5	31.3	30.5	29.2	30.0	31.4
Co-morbidity	Cardiovascular	2377	0.9	1.1	0.9	0.8	0.7	0.8
	Hypertension	13,681	5.0	5.6	5.2	4.5	4.5	5.1
	Diabetes	6919	2.5	2.8	2.7	2.3	2.3	2.6
Percent of the high school completed or more ^2^	<46.6	63,688	23.1	50.7	40.3	11.6	3.0	10.4
	46.6–51.9	68,075	24.7	16.4	40.6	23.7	19.9	22.9
	51.9–55.2	72,227	26.2	32.7	11.0	20.8	39.9	26.5
	>55.2	71,347	25.9	0.2	8.1	43.9	37.2	40.3
Percent of the elderly ^2^ (>65 years)	<4.8	71,231	25.9	14.1	20.3	27.0	47.0	20.4
	4.8–6.0	70,978	25.8	10.6	16.3	38.9	28.3	35.0
	6.0–8.3	69,440	25.2	26.8	20.6	21.9	20.8	36.1
	>8.3	63,688	23.1	48.5	42.8	12.2	4.0	8.5
GRDP ^2,3^ (per 1000 US dollar)	<2,665,207	69,362	25.2	42.8	33.1	13.9	14.4	15.9
	2,665,207–5,145,783	68,853	25.0	24.8	35.2	38.1	14.4	13.8
	5,145,783–10,085,624	99,362	36.1	20.2	17.5	39.6	50.6	57.5
	>10,085,624	37,760	13.7	12.2	14.2	8.5	20.6	12.8
Cause of death	Non-accidental	3796	1.4	1.6	1.6	1.2	1.2	1.3
	Cardiovascular	720	0.3	0.3	0.3	0.2	0.2	0.2
	Cerebrovascular	295	0.1	0.1	0.1	0.1	0.1	0.1
	Respiratory	152	0.1	0.1	0.1	0.1	0.0	0.1
	Cancer	2179	0.8	0.9	0.9	0.7	0.7	0.7
	Lung cancer	461	0.2	0.2	0.2	0.1	0.2	0.1

^1^ NHIS-NSC provided income as percentiles (“The manual for User of the National Health Insurance Service of National Sample Cohort Database”). ^2^ Three area-level characteristics of subjects at baseline in 2002 and theses were categorized by those of quartiles. ^3^ The gross regional domestic product (GRDP, current year) in 2005.

**Table 2:** Adjusted hazard ratios (HRs) and 95% confidence intervals (CIs) of non-accidental and cause-specific mortality for an increase of 10 μg/m^3^ in long-term PM_10_ concentration by four confounder models in 275,337 National Health Insurance Service- National Sample Cohort (NHIS-NSC) subjects for 2002–2013 in South Korea.

Model ^1^	HRs (95% CIs)					
	Non-Accidental	Cardiovascular	Cerebrovascular	Respiratory	Cancer	Lung Cancer
Model 1	0.95 (0.90, 0.99 )	0.88 (0.79, 0.98)	0.89 (0.75, 1.05)	0.96 (0.76, 1.22)	0.97 (0.91, 1.03)	0.86 (0.75, 0.98)
Model 2	0.98 (0.93, 1.03 )	0.91 (0.82, 1.02)	0.93 (0.79, 1.11)	1.05 (0.83, 1.33)	0.98 (0.92, 1.05)	0.88 (0.77, 1.01)
Model 3	0.97 (0.93, 1.02)	0.91 (0.81, 1.01)	0.93 (0.78, 1.10)	1.05 (0.83, 1.32)	0.98 (0.92, 1.05)	0.89 (0.77, 1.01)
Model 4	1.05 (0.99, 1.11)	1.02 (0.90, 1.16)	1.14 (0.93, 1.39)	1.19 (0.91, 1.57)	1.02 (0.95, 1.10)	0.96 (0.82, 1.13)

^1^ Model 1: + sex, age; Model 2: + income, smoking, alcohol use, obese, exercise; Model 3: + comorbidity of cardio-vascular disease, cerebrovascular, and diabetes; Model 4 (primary model): + district-level percent of high school education completed or more, percent of the elderly, and GRDP.

## Supplementary Materials

The following are available online at www.mdpi.com/1660-4601/14/10/1103/s1, Figure S1: Schematic diagram of exclusion criteria and numbers of National Health Insurance Service- National Sample Cohort subjects included or excluded after the application of each criterion, Figure S2: Maps of predicted district-specific annual concentrations of PM_10_ in 2010, South Korea, Table S1: Definition of four exposure assessment (EA) approaches, Equations for individual-level 5-year PM_10_ average concentrations by the four different exposure assessment (EA) approaches, Table S2: Descriptive statistics and correlation coefficients of long-term PM_10_ concentrations of 275,337 National Health Insurance Service- National Sample Cohort (NHIS-NSC) subjects across four exposure assessment (EA) approaches, Table S3: Adjusted hazard ratios (HRs) and 95% confidence intervals (CIs) of non-accidental and cause-specific mortality for an increase of 10 μg/m^3^ in the long-term concentration to PM_10_ defined by four different exposure assessment (EA) approaches, Table S4: Adjusted hazard ratios (HRs) and 95% confidence intervals (CIs) of mortality for an increase of 10 μg/m^3^ in 12-year average PM_10_ concentrations in 275,337 National Health Insurance Service-National Sample Cohort (NHIS-NSC) subjects for 2002–2013 in South Korea, Table S5: Adjusted hazard ratios (HRs) and 95% confidence intervals (CIs) of mortality for an increase of 10 μg/m^3^ in measurement-based long-term PM_10_ concentrations in 237,224 National Health Insurance Service- National Sample Cohort (NHIS-NSC) subjects for 2002–2013 in South Korea, Table S6: Adjusted hazard ratios (HRs) and 95% confidence intervals (CIs) of mortality for an increase of 10 μg/m^3^ in the long-term PM_10_ concentrations from time-dependent Cox proportional hazards models, Table S7: Adjusted hazard ratio (HRs) and 95% confidence interval (CIs) of mortality for an increase of 10 μg/m^3^ in the long-term PM_10_ concentrations from Cox proportional hazards model incorporating random effects for subjects’ residential districts at baseline, Table S8: Summaries of previous cohort studies of the association between long-term exposure to PM_10_ and mortality.

## References

[1] Beelen R., Raaschou-Nielsen O., Stafoggia M., Andersen Z.J., Weinmayr G., Hoffmann B., Wolf K., Samoli E., Fischer P., Nieuwenhuijsen M. (2014). Effects of long-term exposure to air pollution on natural-cause mortality: An analysis of 22 European cohorts within the multicentre ESCAPE project. Lancet.

[2] Hoek G., Krishnan R.M., Beelen R., Peters A., Ostro B., Brunekreef B., Kaufman J.D. (2014). Long-term air pollution exposure and cardio- respiratory mortality: A review. Environ. Health.

[3] Di Q., Wang Y., Zanobetti A., Wang Y., Koutrakis P., Choirat C., Dominici F., Schwartz J.D. (2017). Air pollution and mortality in the Medicare population. N. Engl. J. Med..

[4] Ueda K., Nagasawa S., Nitta H., Miura K., Ueshima H. (2012). Exposure to particulate matter and long-term risk of cardiovascular mortality in Japan: NIPPON DATA80. J. Atheroscler. Thromb..

[5] Cao J., Yang C., Li J., Chen R., Chen B., Gu D., Kan H. (2011). Association between long-term exposure to outdoor air pollution and mortality in China: A cohort study. J. Hazard. Mater..

[6] Zhang P., Dong G., Sun B., Zhang L., Chen X., Ma N., Yu F., Guo H., Huang H., Lee Y.L. (2011). Long-term exposure to ambient air pollution and mortality due to cardiovascular disease and cerebrovascular disease in Shenyang, China. PLoS ONE.

[7] Zhou M., Liu Y., Wang L., Kuang X., Xu X., Kan H. (2014). Particulate air pollution and mortality in a cohort of Chinese men. Environ. Pollut..

[8] Wong C.M., Lai H.K., Tsang H., Thach T.Q., Thomas G.N., Lam K.B.H., Chan K.P., Yang L., Lau A.K.H., Ayres J.G. (2015). Satellite-based estimates of long-term exposure to fine particles and association with mortality in elderly Hong Kong residents. Environ. Health Perspect..

[9] Tseng E., Ho W.C., Lin M.H., Cheng T.J., Chen P.C., Lin H.H. (2015). Chronic exposure to particulate matter and risk of cardiovascular mortality: Cohort study from Taiwan. BMC Public Health.

[10] Katanoda K., Sobue T., Satoh H., Tajima K., Suzuki T., Nakatsuka H., Takezaki T., Nakayama T., Nitta H., Tanabe K. (2011). An association between long-term exposure to ambient air pollution and mortality from lung cancer and respiratory diseases in Japan. J. Epidemiol..

[11] Brauer M., Amann M., Burnett R.T., Cohen A., Dentener F., Ezzati M., Henderson S.B., Krzyzanowski M., Martin R.V., van Dingenen R. (2012). Exposure assessment for estimation of the global burden of disease attributable to outdoor air pollution. Environ. Sci. Technol..

[12] Lim S.S., Vos T., Flaxman A.D., Danaei G., Shibuya K., Adair-Rohani H., AlMarzroa M.A., Amann M., Anderson H.R., Andrews K.G. (2012). A comparative risk assessment of burden of disease and injury attributable to 67 risk factor clusters in 21 regions, 1990–2010: A systematic analysis for the Global Burden of Disease Study 2010. Lancet.

[13] Apte J.S., Marshall J.D., Cohen A.J., Brauer M. (2015). Addressing global mortality from ambient PM_2.5_. Environ. Sci. Technol..

[14] Zhang Q., Jiang X., Tong D., Davis S.J., Zhao H., Geng G., Feng T., Zheng B., Lu Z., Streets D.G. (2017). Transboundary health impacts of transported global air pollution and international trade. Nature.

[15] Krewski D., Jerrett M., Burnett R.T., Ma R., Hughes E., Shi Y., Turner M.C., Pope C.A., Thurston G., Calle E.E. (2009). Extended Follow-Up and Spatial Analysis of the American Cancer Society Study Linking Particulate Air Pollution and Mortality. HEI Research Report 140.

[16] Pope C.A., Burnett R.T., Thun M.J., Calle E.E., Krewski D., Ito K., Thurston G.D. (2002). Lung cancer, cardiopulmonary mortality, and long-term exposure to fine particulate air pollution. JAMA.

[17] Laden F., Schwartz J., Speizer F.E., Dockery D.W. (2006). Reduction in fine particulate air pollution and mortality extended follow-up of the Harvard six cities study. Am. J. Respir. Crit. Care Med..

[18] Lee J., Lee J.S., Park S.H., Shin S.A., Kim K. (2017). Cohort profile: The National Health Insurance Service-National Sample Cohort (NHIS-NSC), South Korea. Int. J. Epidemiol..

[19] Kim S.Y., Song I. (2017). National-scale exposure prediction for long-term concentrations of particulate matter and nitrogen dioxide in South Korea. Environ. Pollut..

[20] Song I., Kim S.Y. (2016). Estimation of representative area-level concentrations of particulate matter (PM_10_) in Seoul, Korea (in Korean). J. Korean Assoc. Geogr. Inf. Stud..

[21] KOSIS. http://kosis.kr/statHtml/statHtml.do?orgId=101&tblId=DT_1INOO02&conn_path=I2.

[22] KOSIS. http://kosis.kr/statHtml/statHtml.do?orgId=202&tblId=DT_F10101&conn_path=I2.

[23] Pope C.A., Dockery D.W. (2006). Health effects of fine particulate air pollution: Lines that connect. J. Air & Waste Manage. Assoc..

[24] U.S. EPA (2009). Integrated Science Assessment for Particulate Matter (Report No. EPA/600/R-08/139F).

[25] NIER (2014). Annual Report of Air Quality in Korea 2014 (Report No. NIER-GP2015-087).

[26] Fischer P.H., Marra M., Ameling C.B., Hoek G., Beelen R., de Hoogh K., Breugelmans O., Kruize H., Janssen N.A., Houthuijs D. (2015). Air pollution and mortality in seven million adults: The Dutch Environmental Longitudinal Study (DUELS). Environ. Health Perspect..

[27] Thurston G.D., Ahn J., Cromar K.R., Shao Y., Reynolds H.R., Jerrett M., Lim C.C., Shanley R., Park Y., Hayes R.B. (2016). Ambient particulate matter air pollution exposure and mortality in the NIH-AARP diet and health cohort. Environ. Health Perspect..

[28] Ostro B., Lipsett M., Reynolds P., Goldberg D., Hertz A., Garcia C., Henderson K.D., Bernstein L. (2010). Long-term exposure to constituents of fine particulate air pollution and mortality: Results from the California Teachers Study. Environ. Health Perspect..

[29] Yi S.J., Kim H., Kim S.Y. (2016). Exploration and application of regulatory PM_10_ measurement data for developing long-term prediction models in South Korea (in Korean). J. Korean Soc. Atmos. Environ..

[30] Crouse D.L., Peters P.A., Hystad P., Brook J.R., van Donkelaar A., Martin R.V., Villeneuve P.J., Jerrett M., Goldberg M.S., Pope C.A. (2015). Ambient PM_2.5_, O_3_ and NO_2_ exposures and associations with mortality over 16 years of follow-up in the Canadian Census Health and Environment Cohort (CanCHEC). Environ. Health Perspect..

[31] Hart J.E., Liao X., Hong B., Puett R.C., Yanosky J.D., Suh H., Kioumourtzoglou M.A., Spiegelman D., Laden F. (2015). The association of long-term exposure to PM_2.5_ on all-cause mortality in the Nurses’ Health Study and the impact of measurement-error correction. Environ. Health.

[32] Pope C.A. (2007). Mortality effects of longer term exposures to fine particulate air pollution: Review of recent epidemiological evidence. Inhal. Toxicol..

[33] Pelucchi C., Negri E., Gallus S., Boffetta P., Tramacere I., la Vechhia C. (2009). Long-term particulate matter exposure and mortality: A review of European epidemiological studies. BMC Public Health.

[34] Dockery D.W., Pope C.A., Xu X., Spengler J.D., Ware J.H., Fay M.E., Ferris B.G., Speizer F.E. (1993). An association between air pollution and mortality in six U.S. cities. N. Engl. J. Med..

[35] Migration Statistics. KOSIS. http://kostat.go.kr/portal/korea/kor_nw/2/2/4/index.board?bmode=read&aSeq=311470.

[36] 2010 Census. KOSIS. http://kostat.go.kr/portal/korea/kor_nw/2/2/1/index.board?bmode=read&aSeq=250515&pageNo=21&rowNum=10&amSeq=&sTarget=&sTxt=.

[37] Carey I.M., Atkinson R.W., Kent A.J., van Staa T., Cook D.G., Andersen H.R. (2013). Mortality associations with long-term exposure to outdoor air pollution in a national English cohort. Am. J. Respir. Crit. Care Med..

[38] Heinrich J., Thiering E., Rzehak P., Krämer U., Hochadel M., Rauchfuss K.M., Gehring U., Wichmann H.E. (2013). Long-term exposure to NO_2_ and PM_10_ and all-cause and cause-specific mortality in a prospective cohort of women. Occup. Environ. Med..

[39] Szpiro A.A., Sheppard L., Lumley T. (2011). Efficient measurement error correction with spatially misaligned data. Biostatistics.

