# Supplementary material for: Association between Long-Term Exposure to Particulate Matter Air Pollution and Mortality in a South Korean National Cohort: Comparison across Different Exposure Assessment Approaches
Source: Int J Environ Res Public Health. 2017 Sep 23;14(10):1103. doi: 10.3390/ijerph14101103 (PMC5664604; doi:10.3390/ijerph14101103)
Supplement: Supplementary file 1 [file ijerph-14-01103-s001.pdf]

International Journal of
Environmental Research and Public Health

Supplementary Materials: Association between long-term exposure to particulate matter air pollution and mortality in a South Korean national cohort: comparison across different exposure assessment approaches

Okjin Kim, Sun-Young Kim *, Ho Kim *

Supplementary tables and figures

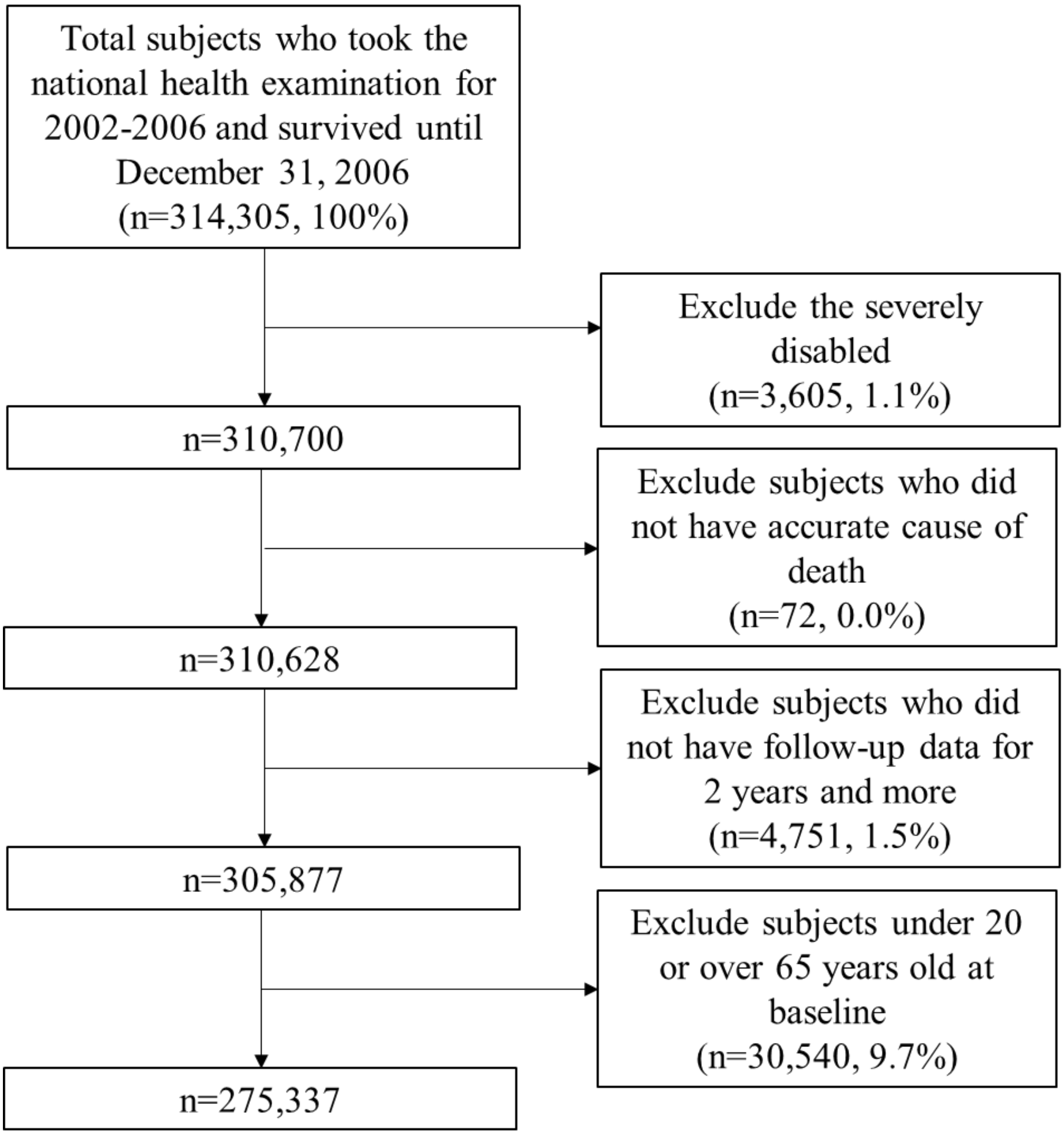

Figure S1. Schematic diagram of exclusion criteria and numbers of National Health Insurance Service- National Sample Cohort subjects included or excluded after the application of each criterion.

International Journal of
Environmental Research and Public Health

Table S1. Definition of four exposure assessment (EA) approaches.

EA	Time-varying exposure	Residential mobility
EA1	O	O
EA2	O	X
EA3	X	O
EA4	X	X

Equations for individual-level 5-year PM_{10} average concentrations by the four different exposure assessment (EA) approaches.

PM_{t,s_t} : PM_{10} predicted annual average concentration of each person at address s_t in year t.

t : 2002 to 2006

t^* : baseline year (2002)

s^* : address in baseline year (2002)

$$EA1 = \frac{1}{5}\sum_{t=2002}^{2006} PM_{t,s_t}$$

$$EA2 = \frac{1}{5}\sum_{t=2002}^{2006} PM_{t,s^*}$$

$$EA3 = \frac{1}{5}\sum_{t=2002}^{2006} PM_{t^*,s_t}$$

$$EA4 = \frac{1}{5}\sum_{t=2002}^{2006} PM_{t^*,s^*} = PM_{t^*,s^*}$$

Table S2. Descriptive statistics and correlation coefficients of long-term PM_{10} concentrations of 275,337 National Health Insurance Service- National Sample Cohort (NHIS-NSC) subjects across four exposure assessment (EA) approaches.

EA	PM_{10} concentration (μg/m^3)				Correlation coefficient			
	Mean	SD	Min	Max	EA1	EA2	EA3	EA4
EA1	56.0	6.5	39.2	72.1	1.00			
EA2	55.9	6.6	39.2	71.2	0.96	1.00		
EA3	57.6	8.9	28.1	80.2	0.90	0.87	1.00	
EA4	57.6	9.2	28.1	80.2	0.87	0.91	0.95	1.00

International Journal of
Environmental Research and Public Health

Table S3. Adjusted hazard ratios (HRs) and 95% confidence intervals (CIs) of non-accidental and cause-specific mortality for an increase of 10 $\mu g/m^3$ in the long-term concentration to PM_{10} defined by four different exposure assessment (EA) approaches.

**EA[2]**	**HRs[1] (95% CIs)**					
	**Non-accidental**	**Cardiovascular**	**Cerebrovascular**	**Respiratory**	**Cancer**	**Lung cancer**
EA1	1.05 (0.99,1.11)	1.02 (0.90,1.16)	1.14 (0.93,1.39)	1.19 (0.91,1.57)	1.02 (0.95,1.10)	0.96 (0.82,1.13)
EA2	1.05 (0.99,1.11)	1.02 (0.90,1.16)	1.12 (0.92,1.37)	1.23 (0.93,1.63)	1.02 (0.94,1.10)	0.96 (0.81,1.13)
EA3	1.00 (0.96,1.04)	0.99 (0.90,1.08)	1.07 (0.93,1.23)	1.05 (0.87,1.27)	0.98 (0.93,1.03)	0.92 (0.82,1.04)
EA4	1.00 (0.97,1.05)	1.00 (0.91,1.09)	1.08 (0.94,1.24)	1.05 (0.87,1.27)	0.99 (0.94,1.04)	0.94 (0.84,1.05)

[1] Effect estimates from our primary model (model 4) adjusting for sex, age, income, smoking, alcohol use, obese, exercise, and co-morbidity of cardiovascular disease, cerebrovascular disease, and diabetes, district-level percent of high school education completed or more, percent of the elderly, and gross regional domestic product.

[2] EA1: prediction and address in each year
EA2: prediction in each year and address at baseline

EA3: prediction at baseline and address in each year
EA4: prediction and address at baseline

International Journal of
Environmental Research and Public Health

Table S4. Adjusted hazard ratios (HRs) and 95% confidence intervals (CIs) of mortality for an increase of 10 $\mu g/m^3$ in 12-year average PM_{10} concentrations[1] in 275,337 National Health Insurance Service- National Sample Cohort (NHIS-NSC) subjects for 2002-2013 in South Korea.

**Cause of mortality**	**HRs[2] (95% CIs)**
Non-accidental	1.07 (1.00,1.15)
Cardiovascular	1.04 (0.89,1.22)
Cerebrovascular	1.20 (0.94,1.54)
Respiratory	1.26 (0.90,1.77)
Cancer	1.04 (0.95,1.15)
Lung cancer	1.00 (0.81,1.22)

[1] 12-years average concentrations of PM_{10} at addresses at baseline in 2002.
[2] Adjusting for sex, age, income, smoking, alcohol use, obese, exercise, and co-morbidity of cardiovascular disease, cerebrovascular disease, and diabetes, district-level percent of high school education completed or more, percent of the elderly, and gross regional domestic product.

Table S5. Adjusted hazard ratios (HRs) and 95% confidence intervals (CIs) of mortality for an increase of 10 $\mu g/m^3$ in measurement-based long-term PM_{10} concentrations in 237,224[2] National Health Insurance Service- National Sample Cohort (NHIS-NSC) subjects for 2002-2013 in South Korea.

**Cause of mortality**	**HRs[1] (95% CIs)**	
	**Measurement-based PM_{10}**	**EA1[2]**
Non-accidental	1.04 (0.99,1.08)	1.07 (1.00,1.14)
Cardiovascular	1.02 (0.92,1.13)	1.05 (0.91,1.22)
Cerebrovascular	1.05 (0.90,1.24)	1.10 (0.87,1.39)
Respiratory	0.98 (0.79,1.22)	1.05 (0.76,1.45)
Cancer	1.01 (0.95,1.07)	1.02 (0.94,1.11)
Lung cancer	0.98 (0.86,1.12)	0.98 (0.81,1.19)

[1] Adjusting for sex, age, income, smoking, alcohol use, obese, exercise, and co-morbidity of cardiovascular disease, cerebrovascular disease, and diabetes, district-level percent of high school education completed or more, percent of the elderly, and gross regional domestic product.
[2] Subjects who lived in the districts where there are regulatory air pollution monitoring sites.

Table S6. Adjusted hazard ratios (HRs) and 95% confidence intervals (CIs) of mortality for an increase of 10 $\mu g/m^3$ in the long-term PM_{10} concentrations from time-dependent Cox proportional hazards models.

**Cause of mortality**	**HRs[1] (95% CIs)**
Non-accidental	1.03 (0.96 ,1.11)
Cardiovascular	0.91 (0.77,1.06)
Cerebrovascular	1.07 (0.84,1.36)
Respiratory	1.01 (0.71,1.45)
Cancer	1.05 (0.96,1.16)
Lung cancer	1.00 (0.81,1.22)

[1] Adjusting for sex, age, income, smoking, alcohol use, obese, exercise, and co-morbidity of cardiovascular disease, cerebrovascular disease, and diabetes, district-level percent of high school education completed or more, percent of the elderly, and gross regional domestic product.

Table S7. Adjusted hazard ratio (HRs) and 95% confidence interval (CIs) of mortality for an increase of 10 μg/m^3 in the long-term PM_{10} concentrations from Cox proportional hazards model incorporating random effects for subjects' residential districts at baseline.

**Cause of mortality**	**HRs[1] (95% CIs)**
Non-accidental	1.05 (0.99, 1.11)
Cardiovascular	1.02 (0.90, 1.16)
Cerebrovascular	1.14 (0.93, 1.39)
Respiratory	1.19 (0.89, 1.58)
Cancer	1.02 (0.95, 1.10)
Lung cancer	0.96 (0.82, 1.13)

[1] Adjusting for sex, age, income, smoking, alcohol use, obese, exercise, and co-morbidity of cardiovascular disease, cerebrovascular disease, and diabetes, district-level percent of high school education completed or more, percent of the elderly, and gross regional domestic product.

International Journal of
Environmental Research and Public Health

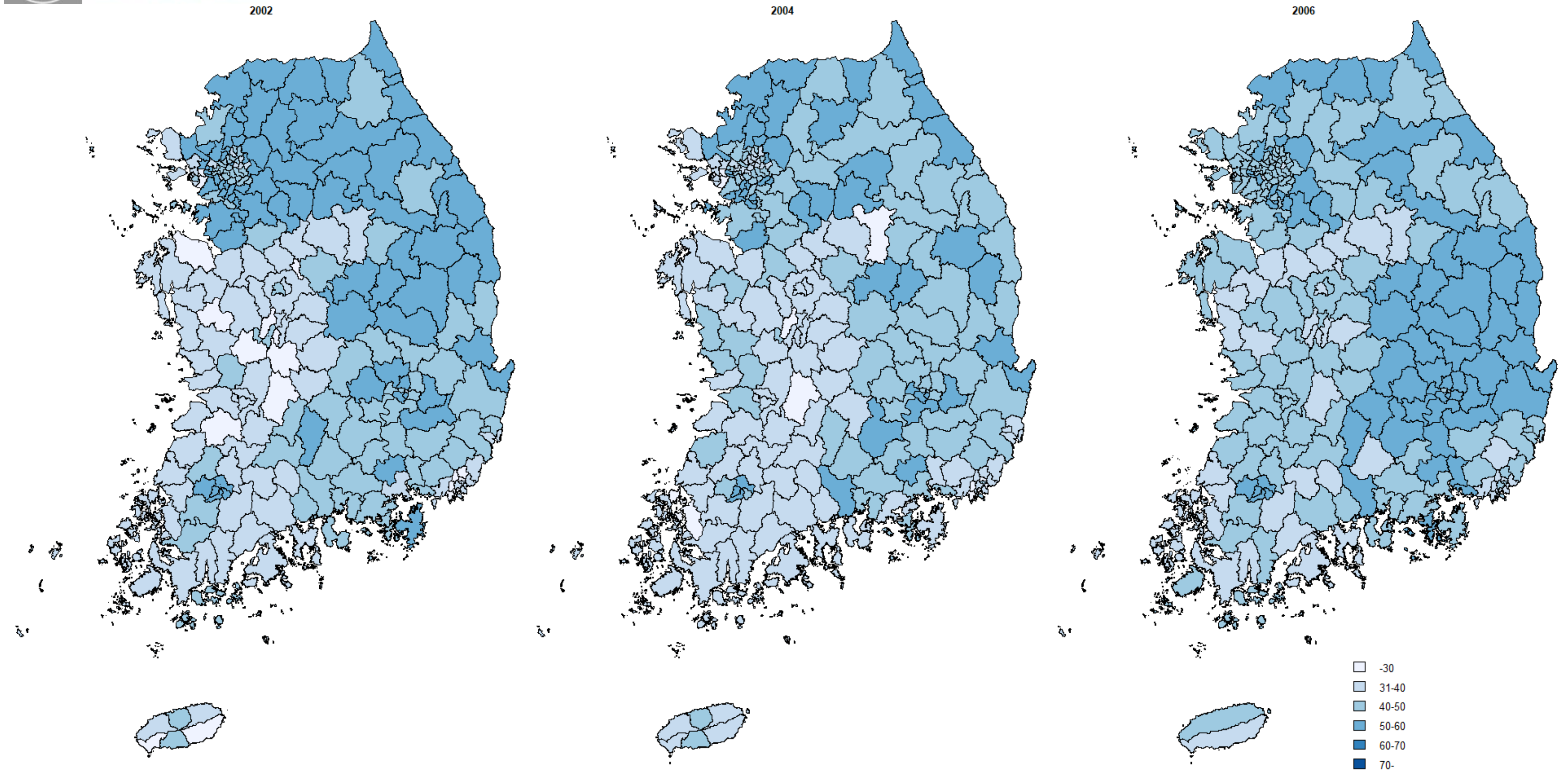

Figure S2. Maps of predicted district-specific annual concentrations of PM_{10} in 2010, South Korea.

Table S8. Summaries of previous cohort studies of the association between long-term exposure to PM_{10} and mortality.

Study	Cohort	N of subjects	Measurements/ predictions	Time	Space	Percent change in mortality risk (95% confidence interval per 10 μg/m³ increase in PM_{10}				
						All-causes	Non-accidental	Cardiovascular *[cerebrovascular]*	Respiratory	Lung cancer
Ueda et al, 2011 [1]	Japan ≥ 30 years nationwide	7,250	Measurements	24 year average	District	-2 (-8, 4)		-14 (-26, 1)		
Fisher et al, 2015 [2]	Netherland, ≥ 30 years, nationwide	7,218,363	Predictions	1 year average	100m x 100m grid		8 (7, 9)	6 (4, 8)	13 (10, 17)	26 (21, 30)
Zhang et al, 2011 [3]	China 35-103 years, 5 districts	9,941	Measurements	1 year average	District			55 (51, 60)		
Zhou et al, 2014 [4]	China ≥ 40 years 25 urban cities	71,431	Measurements	10 year average	City	0.4 (-0.1, 1)		1 (0.4, 1.7)	-0.2 (-0.9, 0.6)	0.3 (-0.2, 0.9)
Carey et al, 2013 [5]	England 40-89 years nationwide	835,607	Predictions	1 year average	1kmx1km grid	7 (-1, 16)		7 (-0.3, 18)	42 (29, 55)	7 (0, 14)
Bentayeb et al, 2015 [6]	France average 43.7 ages nationwide	20,327	Predictions	20 year average	zip code area		17 (0, 37)	17 (-26, 84)	0 (-32, 48)	
Heinrich et al, 2013 [7]	German, ≥ 55 years 10 areas in 7 cities	4,750	Measurements	1 year average	geocoded address		22 (6, 41)	60 (25, 105)	-6 (-52, 85)	139 (34, 325)
Beelen et al, 2014 [8]	Europe ESCAPE cohort	367,251	Predictions	2.5 year average			4 (0, 9)			
Beelen et al ,2014 [9]	Europe, ESCAPE cohort	367,383	Predictions	2.5 year average				2 (-8, 14) *[22 (-9, 63)]*		

1. Ueda, K.; Nagasawa, S.; Nitta, H.; Miura, K.; Ueshima, H. Exposure to particulate matter and long-term risk of cardiovascular mortality in Japan: NIPPON DATA80. *J. Atheroscler. Thromb.* **2012**, *19*, 246–254.
2. Crouse, D.L.; Peters, P.A.; Hystad, P.; Brook, J.R.; van Donkelaar, A.; Martin, R.V.; Villeneuve, P.J.; Jerrett, M.; Goldberg, M.S.; Pope, C.A. III; et al. Ambient $PM_{2.5}$, O_3 and NO_2 exposures and associations with mortality over 16 years of follow-up in the Canadian Census Health and Environment Cohort (CanCHEC). *Environ. Health Persp.* **2015**, *123*, 1180–1186.
3. Zhang, P.; Dong, G.; Sun, B.; Zhang, L.; Chen, X.; Ma, N.; Yu, F.; Guo, H.; Huang, H.; Lee, Y.L.; et al. Long-term exposure to ambient air pollution and mortality due to cardiovascular disease and cerebrovascular disease in Shenyang, China. *PLoS ONE* **2011**, 6, e20827.
4. Zhou, M.; Liu, Y.; Wang, L.; Kuang, X.; Xu, X.; Kan, H. Particulate air pollution and mortality in a cohort of Chinese men. *Environ. Pollut.* **2014**, *6*, 1–8.
5. 2010 Census. KOSIS. Available online, http://kostat.go.kr/portal/korea/kor_nw/2/2/1/index.board?bmode=read&aSeq=250515&pageNo=21&rowNum=10&amSeq=&sTarget=&sTxt= (assessed on 25 July 2017).
6. Bentayeb M.; Wagner V.; Stempfelet M.; Zins M.; Goldberg M.; Pascal M.; Larrieu S.; Veaudeau P.; Cassadou S.; Eilstein D.; et al. Association between long-term exposure to air pollution and mortality in France: A 25-year follow-up study. *Environ. Int*. **2015**, *85*; 5-14
7. Carey I.M.; Atkinson R.W.; Kent A.J.; van Staa T.; Cook D.G.; Andersen H.R. Mortality associations with long-term exposure to outdoor air pollution in a national English cohort. Am. *J. Resp. Crit. Care* **2013**, *187*, 1226-1233.
8. Beelen, R.; Raaschou-Nielsen, O.; Stafoggia, M.; Andersen, Z.J.; Weinmayr, G.; Hoffmann, B.; Wolf, K.; Samoli, E.; Fischer, P.; Nieuwenhuijsen, M.; et al. Effects of long-term exposure to air pollution on natural-cause mortality: an analysis of 22 European cohorts within the multicentre ESCAPE project. *Lancet* **2014 a**, *383*, 785–795.
9. Beelen R.; Stafoggia M.; Raaschou-Nielsen O.; Andersen Z.J.; Xun W.W.; Katsouyanni K.; Diamakopoulou K.; Brunekreef B.; Weinmayr G.; Hoffmann B.; et al. Long-term exposure to air pollution and cardiovascular mortality, an analysis of 22 European cohorts. *Epidemiology* **2014 b**, *25*, 368-378.

© 2016 by the authors; licensee MDPI, Basel, Switzerland. This article is an open access article distributed under the terms and conditions of the Creative Commons by Attribution (CC-BY) license (http://creativecommons.org/licenses/by/4.0/).
